# The Effect of the Antimicrobial Peptide Plectasin on the Growth Performance, Intestinal Health, and Immune Function of Yellow-Feathered Chickens

**DOI:** 10.3389/fvets.2021.688611

**Published:** 2021-06-23

**Authors:** Xinheng Zhang, Qiqi Zhao, Lijun Wen, Che Wu, Ziqi Yao, Zhuanqiang Yan, Ruoying Li, Liyi Chen, Feiyang Chen, Zi Xie, Feng Chen, Qingmei Xie

**Affiliations:** ^1^Lingnan Guangdong Laboratory of Modern Agriculture, College of Animal Science, South China Agricultural University, Guangzhou, China; ^2^Guangdong Provincial Key Lab of Agro-Animal Genomics and Molecular Breeding & Key Laboratory of Chicken Genetics, Breeding and Reproduction, Ministry of Agriculture, Guangzhou, China; ^3^Guangdong Engineering Research Center for Vector Vaccine of Animal Virus, Guangzhou, China; ^4^South China Collaborative Innovation Center for Poultry Disease Control and Product Safety, Guangzhou, China; ^5^Guangdong Hinabiotech Co., Ltd, Guangzhou, China; ^6^Guangdong Enterprise Key Laboratory for Animal Health and Environmental Control, Wen's Foodstuff Group Co., Ltd, Yunfu, China

**Keywords:** plectasin, performance, immune, intestinal health, yellow-feathered chickens

## Abstract

The goal of the study was to test the effects of an antibiotic substitute, plectasin, on the growth performance, immune function, intestinal morphology and structure, intestinal microflora, ileal mucosal layer construction and tight junctions, ileal immune-related cytokines, and blood biochemical indices of yellow-feathered chickens. A total of 1,500 one-day-old yellow-feathered chicks were randomly divided into four dietary treatment groups with five replicates in each group and 75 yellow-feathered chicks in each replication, as follows: basal diet (group A); basal diet supplemented with 10 mg enramycin/kg of diet (group B), basal diet supplemented with 100 mg plectasin/kg of diet (group C), and basal diet supplemented with 200 mg plectasin/kg of diet (group D). It was found that the dietary antimicrobial peptide plectasin could improve the ADG and had better F/G for the overall period of 1–63 days. Dietary plectasin can enhance H9N2 avian influenza virus (AIV) and Newcastle disease virus (NDV) antibody levels of yellow-feathered chickens at 21, and 35 days of age. Dietary plectasin can enhance the intestine structure, inhibit *Escherichia coli* and proinflammatory cytokines in the ileum, and ameliorate the blood biochemical indices of yellow-feathered chickens at 21 days of age. This study indicates that the antimicrobial peptide plectasin has beneficial effects on the growth performance, intestinal health and immune function of yellow-feathered chickens.

## Introduction

Antibiotics have been used by the poultry industry to enhance the health and productivity of flocks and treat diseases (1, 2). However, the long-term use of antibiotics will induce pathogens to produce resistance genes and reduce the bactericidal effect of antibiotics on the pathogens (3, 4). Due to the large amounts of antibiotics remaining in the bodies of livestock and poultry, they will eventually enter the human body through meat, eggs, milk, and other livestock and poultry products (5). Over time, antibiotics will accumulate in the human body, causing various organ diseases and immunosuppression (6). Furthermore, the wide use of antibiotics as a therapeutic measure frequently causes disturbances in the human gut microbiota (7). The use of antimicrobials is strictly regulated by the Food and Drug Administration (FDA) and the USDA to warrant their safety and efficacy (2). The limitations of antibiotics have led to reductions in animal performance and feed conversion and a rise in the incidence of certain animal diseases; thus, there is an urgent need to explore alternatives to antibiotics (8).

Antimicrobial peptides (AMPs) are small molecules (12–15 amino acids) containing positive charges and amphipathic structures, which have broad-spectrum antibacterial, fungal, and protozoan activity as well as a certain level of virus activity (9). Due to the broad spectrum of antimicrobial activity and low propensity for the development of bacterial resistance, AMPs have also been regarded as important alternatives to antibiotics (10). AMPs have been reported that have beneficial effects on growth performance, intestinal microflora and morphology, immunity function and nutrient digestibility in chickens (11, 12). AMPs are innate and specific immune molecules that regulate pro-inflammatory and anti-inflammatory reactions and chemotactic activity and improve specific immunity (13). According to the final effect of an AMP on target cells, its mechanism can be divided into two types: the first is membrane decomposing peptides that cause cell death by changing the permeability of cell membranes (14) and the second is a non-membrane active peptide that affects intracellular targets and interferes with cell metabolism to show its bactericidal activity (15). It has been reported that dietary AMPs can improve the intestinal tissue structure and promote growth (16–19).

Defensins are cationic antibacterial active peptides that are found in antibacterial peptides, which are widely present in animals and plants (20, 21). Plectasin was the first fungal defensin to be isolated from the saprophytic ascomycete Pseudoplectania nigrella (22). Plectasin contains 40 amino acids (GFGCN GPWNE DDLRC HNHCK SIKGY KGGYC AKGGF VCKCY) that has a molecular weight of about 4.4 ku, and has demonstrated activity against bacteria, such as gram-negative and gram-positive, in particular various strains resistant to conventional antibiotics (22, 23). Plectasin contains α-β motif that is comprised of an α-helix and two antiparallel β-strands, and is stabilized by three disulphide bonds (Cys4-Cys30, Cys15-Cys37 and Cys19-Cys39) (22). Cysteine-rich peptide is a broad-spectrum antimicrobial agent, the unique reactivity of the cysteine side chain makes it possible to carry out a variety of useful chemical reactions to regulate innate immune systems in different life forms such as bacteria, protozoa, fungi, plants, insects and animals (24, 25). The range of Minimum Inhibitory Concentration (MIC) of plectasin was between 0.007 mg/ml to 1.8 mg/ml (26). Plectasin was confirmed to promote the proliferation of beneficial intestinal bacteria and have no cytotoxicity to normal human bronchial epithelial cell, A549 cells or lung fibroblasts, and did not induce IL-8 transcription or production in A549 cells (27). In 2010, it was reported that plectasin acts antibacterially by targeting the bacterial cell wall precursor lipid II (28). In 2015, plectasin was the first-reported animal toxin-like fungal defensin with the ability to block potassium channels through recognizing the channel pore region (29). In 2017, Li et al. (30) reported the research advances on plectasin and its derivatives as new potential antimicrobial candidates. One report showed that dietary plectasin have beneficial effects on broilers (Arbor Acres, white-feathered chickens) (23), however, there has been no report on the effect of the antimicrobial peptide plectasin on yellow-feathered chickens. Hence, this study aims to investigate the effects of dietary plectasin on the growth performance, intestinal health, and immune function of yellow-feathered chickens. The significance of this study is that it explores the mechanism by which plectasin can act as a feed additive for yellow-feathered chickens and provides a certain theoretical basis for the replacement of antibiotics by plectasin.

## Materials and Methods

### Animals, Plectasin, and Experimental Design

Fifteen hundred healthy 1-day-old yellow-feathered chicks with similar body weights were purchased from Guangdong Wen's Food Group Co., Ltd., China. The plectasin (amino acid sequence: GFGCNGPWNEDDLRCHNHCKSIKGYKGGYCAKGGFVCKCY; NCBI accession number: PDB: 6K50_A) used in this study was purchased from Guangdong Hinabiotech Co., Ltd. (Guangzhou, China). The method for producing the plectasin was as follows according to previous reported study with some modifications (31): The mycelium gene from saprophytic ascomycetes was cloned into vector pPICZ αA to construct a recombinant plasmid. The recombinant plasmid was transferred into Pichia pastoris by electroporation. The positive transformants were cultured in 10 mL yeast extract peptone glucose (YPD) medium containing 100 μg/mL bleomycin at 30°C and 250 r/min for 24 h. After that, the positive transformants were centrifuged at 4°C and 4,000 r/min for 5 min. The supernatant was discarded and the bacteria were re-suspended with 1 L YPD culture medium. Finally, recombinant mycelium was prepared by high-density fermentation in a 2 T fermentor containing basic salt medium (BSM), in which the dissolved oxygen was kept above 30%. The original carbon source in the culture medium was consumed, and then the expression was induced by methanol at 37°C for 72 hours, the concentration of total protein in the supernatant was 4 g/L. The yeast cells were removed by centrifugation, the supernatant of the fermentation broth was concentrated, and then purified by ion exchange chromatography, finally, the recombinant plectasin was obtained.

A single-factor, randomized trial design was used in this study. The 1,500 one-day-old yellow-feathered chicks were randomly divided into four treatment groups with five replicates in each group and 75 yellow-feathered chicks in each replication. All of the chickens were reard in an over pressure isolator and the temperature of the isolator was maintained 32°C during the first week, and the it was reduced 2°C each week until reaching at 22°C in week 4, and then the temperature was lasted to the end of the experiment. The light was programmed as continuous for 24 h for the first 2 days, while chickens were exposed to 23 h of white light a day and 1 h a dark after 2 days. Plastic drinkers and feeders were used for supplying water and feed. All of chickens in different treatment groups were free to feed intake and water every day. The overall trial period was 63 days. The groupings are shown in Table 1. The basal diet was feed based on corn-soybean, manufactured by Guangdong Wen's Food Group Co., Ltd., designed based on the “NY/T33-2004 feeding standard of chicken” and the recommend nutrition standard for yellow-feathered chickens. Table 2 shows the composition and nutritional information for the basal diet.

**Table 1:** The grouping of experiment.

**Group**	**Treatment**	**Quantity**
A	Basal diet	375
B	Basal diet + 10 mg enramycin/kg diet	375
C	Basal diet + 100 mg plectasin/kg diet	375
D	Basal diet + 200 mg plectasin /kg diet	375

**Table 2:** The composition and nutrition level of basal diet (air-dry basis).

**Raw material (kg)**	**Day 1–21**	**Day 22–42**	**Day 43–63**	**Nutrition level^b^**	**Day 1–21**	**Day 22–42**	**Day 43–63**
Corn	61.643	64.864	68.067	Metabolic energy/ (MJ/kg)	12.13	12.76	13.18
Soybean	30.250	26.554	21.731	Crude protein (%)	20.50	18.50	17.00
Corn protein powder	3.000	1.507	2.437	Crude fat (%)	3.66	5.71	6.78
Soybean oil	1.050	3.120	4.090	Crude fiber (%)	2.23	2.12	1.98
Calcium hydrophosphate	1.280	1.070	0.920	Ca (%)	0.90	0.85	0.80
Limestone	1.290	1.300	1.300	Total *P* (%)	0.58	0.53	0.48
Salt	0.340	0.350	0.350	Available *p* (%)	0.35	0.31	0.28
Mold inhibitor	0.100	0.100	0.100	Lys (%)	1.21	1.10	0.97
Choline chloride	0.080	0.080	0.080	Digestible Lys (%)	1.10	1.00	0.88
Premix^a^	0.400	0.400	0.400	Digestible Met + Digestible Cys (%)	0.75	0.78	0.65
Lys	0.342	0.317	0.308				
DL-Met	0.170	0.267	0.163				
Thr	0.55	0.070	0.054				
Total	100.00	100.00	100.00				

a *The premix provided the following per kg of diets: Cu 11 mg, Fe 149 mg, Mn 32 mg, Zn 35 mg, I 0.50 mg, Se 0.35 mg, VA 15 000 IU, VD 33 000 IU, VE 46 mg, VB1 7 mg, VB2 11 mg, VB6 14 mg, VB12 30 ug, niacin 83 mg, D- Pantothenic acid 32 mg, folic acid 2 mg, biotin 190 ug*.

b *The value of metabolizable energy is calculated, other items are tested*.

### Production Performance

The weight of each yellow-feathered chick and the feed consumption of each treatment group were recorded daily. The average daily feed intake (ADFI), average daily gain (ADG), feed to gain ratio (F/G). and the survival rate of chickens at 1–21, 22–42, 43–63 and 1–63 days of age were evaluated.

### Determination of Antibody Titer

The yellow-feathered chickens were vaccinated with the commercial inactivated vaccines for H9N2 avian influenza virus (AIV) (cat. No. DL034, Beijing biolab technology co. LTD) and Newcastle disease virus (NDV) (cat. no. DL032, Beijing biolab technology co. LTD) via spray immunization at 1-day-old, and then they were revaccinated with the same vaccine via subcutaneous neck injection at 10 days of age. There were 25 birds in each treatment group with five chickens were randomly selected from each replicate (total five replicates) at 7, 21, 35, and 42 days old. Five milliliters of blood was transferred into the centrifuge tube by a 10 mL syringe, left to stand for 30 min at room temperature, and centrifuged for 10 min at 3,000 rpm. Then, the supernatant was collected to detect the antibody levels of H9N2 AIV and NDV by HI assay as described previously (32, 33).

### Observation and Measurement of Intestinal Morphology

Chickens at 21 days of age were selected for testing the intestinal morphology (34). There were 25 birds in each treatment group with five chickens were randomly selected from each replicate (total five replicates) for euthanasia by cervical dislocation at 21 days of age, and 1 cm gut tissue samples from the duodenum, jejunum, and ileum were taken, the gut cavity was carefully washed with PBS (phosphate buffer saline) buffer, and samples were put into 5 mL centrifuge tubes with 10% formalin solution to fix samples. The hematoxylin-eosin staining and paraffin section making were conducted by using the fixed samples. The villus lengths and crypt depths of ten intact villi were measured, and calculated the average values of each tissue.

### Detection of Immune-Related Cytokines, Mucosal Layer Structure, and Tight-Junction-Related Factors of Ileum Using Quantitative Real-Time PCR (qRT-PCR)

Chickens at 21 days of age were selected for testing the immune-related cytokines, the ileum mucosal layer structure, and tight-junction-related factors (35, 36). There were 25 birds in each treatment group with five chickens were randomly selected from each replicate (total five replicates) for euthanasia by cervical dislocation at 21 days of age. A small piece of the ileum was cut off in an aseptic environment and put into a 2 mL EP tube containing 1 mL TRIzol (Invitrogen, Carlsbad, CA, USA). Total RNA was extracted from the samples with TRIzol and a NanoDrop-2000 spectrophotometer (ThermoFisher Scientific Co., Waltham, MA, USA) with the 260/280 nm absorbance ratio was used to measure the purity and concentration of the total RNA. The RecerTra Ace qPCR RT Master Mix with gDNA remover (TOYOBO Co., LTD. Life Science Department, OSAKA, Japan) was used for reverse transcription of cDNA with 1.0 μg RNA of each sample. Quantitative Real-time PCR (qRT-PCR) was performed in triple with the following reaction system: nuclease free water (1.5 μL), 1 μL of each forward and reverse primer of each gene (5 μM), 1.5 μL of cDNA (1:20 dilution), and the SYBR® Premix Ex Taq^TM^ II kit (ThermoFisher Scientific Co., Waltham, MA, USA) was 5 μL, the reaction performed on an CFX96 Touch™ real-time PCR detection system (Bio-Rad Laboratories, Mississauga, ON, Canada). The specific primers used for the mucosal layer structure and the tight-junction-related factors and inflammation-related cytokines of ileum were designed using Primer Express 3.0 (Applied Biosystems) in accordance with the sequences published in GenBank for qRT-PCR and are shown in Table 3. The PCR condition was this: initial denaturation (95°C for 3 min), followed by 40 cycles (95°C for 5 s and at 60°C for 30 s). Each sample was analyzed in triplicate. The specificity of the PCR products was evaluated by melting curve analysis. The internal reference gene was *GAPDH*. The 2^−ΔΔCt^ method was used to calculate the relative expression of each gene (37).

**Table 3:** Primers for qRT-PCR detection of ileum mucosal barrier related indices and inflammation-related cytokines.

**Gene names**	**Primers**	**Accession No**.
*ZO-1*	F: GCACAAGGAGGTCAGCCAGATG	Accession: XM_015278981.2
	R: ATCATTGCCACCAGCGAGCC	
*Claudin-3*	F: CTTCATCGGCAACAACATC	Accession: NM_204202.1
	R: CATGGAGTCGTACACCTTG	
*TFF2*	F: CCAGGAATCTCAGCAGCAGACT	Accession: XM_416743.4
	R: AGCCACAGTTCACTCGGATACG	
*MUC2*	F: CGCAAGTCCTGGGCAGAGAAAG	Accession: NM_001318434.1
	R: GCAGCAACAGCAGAACAGAAGC	
*IFN-γ*	F: GCCTCCACACCTTCCTCCAAGA	Accession: NM_205427.1
	R: GCGTGTTGCCTGTGAGGTTGT	
*IFN-α*	F: CTGCCTCCACACCTTCCTCCAA	Accession: NM_205427.1
	R: GCGTGTTGCCTGTGAGGTTGT	
*IL-1β*	F: AGCAGCAGCCTCAGCGAAGA	Accession: XM_015297469.1
	R: CCAGCCCTCCCATCCTTACCTT	
*IL-17A*	F: AATCGGTCTCTCGCTCCTTGGA	Accession: XM_426223.6
	R: GGCACTCGGCATCAGCAATCA	
*IL-22*	F: AGGGAGAACAACCGCTGCTACA	Accession: NM_001199614.1
	R: GAGGTCAGGGATGCCAGGAACT	
*IL-6*	F: GTGTGCGAGAACAGCATGGAGA	Accession: NM_204628.1
	R: CTGGAGAGCTTCGTCAGGCATT	
*GAPDH*	F: GCTGTGGAGAGATGGCAGAGGT	Accession: NM_204305.1
	R: ACGGCAGGTCAGGTCAACAACA	

### Intestinal Flora Determination

Chickens at 21 days of age were selected for testing the intestinal flora (38). There were 25 birds in each treatment group with five chickens were randomly selected from each replicate (total 5 replicates) for euthanasia by cervical dislocation at 21 days of age. One hundred milligram ileum and cecum samples under sterilization were put into 2 mL sterilizing centrifuge tubes and stored at −80°C after pre-freezing in liquid nitrogen. *Escherichia coli* and *Lactobacilli* were detected in the ileum and cecum by the absolute quantitative PCR method. Gut microorganism DNA were extracted with the feces genome DNA Extracting Kit [DP328, Tiangen Biotech (Beijing) Co. Ltd.]. Primers (*E. coli*: F: 5′- GTATTGACCCGTCAGGTGTG−3′ R: 5′- GCTGGCCTGTTTGGTGATAA-3′; *Lactobacilli*: F: 5′- AGATGTTGGGTTAAGTCCCGC-3′ R: 5′- GTGCTGATCCGCGATTACTA-3′) were used to amplify the fragment inserts of *E. coli* (243 bp) and *Lactobacilli* (200 bp) by PCR. DNA standards were prepared from *E. coli* or *Lactobacilli* strains carrying plasmids with *E. coli* or *Lactobacilli* fragment inserts which were ligated into a PMD19-T vector (TaKaRa, Biotechnology, Dalian, China). The DNA concentration was calculated by measuring the absorbance at 260 nm. Using the DNA concentration, the copy number of the plasmid was calculated using the following formula: copy/μL = 6.02 × 10^23^(copy/mol) × DNA concentration (g/μL)/MW (g/mol). Serial 10 fold dilutions from 10^4^ to 10^10^ copies of the purified plasmid were prepared in duplicate to produce a standard curve. The quantitative Real-time PCR (qRT-PCR) was used to evaluate the copy number of *E. coli* and *Lactobacilli* in ileum and cecum with following reaction system: DNA (1.0 μL), 10.0 μL of SYBR Green qPCR Mix (Roche Diagnostics, Shanghai, China), each primer was 0.5 μL, and 8 μL of H_2_O. Samples were amplified under the following conditions: 94°C for 2 min (initial denaturation), 40 cycles of 94°C for 10 s (heat denaturation), 60°C for 40 s (primer annealing). Fluorescence signal acquisition was set at 60°C. The primers for the amplification of *E. coli* by qRT-PCR were: 5′-GTTAATACCTTTGCTCATTGA-3′ and 5′-ACCAGGGTATCTTAATCCTGTT-3′. The primers for *Lactobacilli* amplification by qRT-PCR were as follows: 5′- CCCTTGTCATTAGTTGCCAGCATTAAG-3′ and 5′- CCTCCTCGTTGTACTGTCCATTGTAGC-3′.

### Detection of Blood Biochemical Indices

Chickens at 21 days of age were selected for testing the blood biochemical indices (35). Venous blood was taken from 25 birds in each treatment group with five chickens were randomly selected from each replicate (total five replicates) at 21 days of age. The blood without anticoagulant treatment was put in a water bath at 37°C for 30 min. The serum was isolated after centrifugation at 3,000 r/min for 10 min. Serum biochemical indices were assessed in terms of triglycerides, blood glucose, high-density cholesterol, total cholesterol, albumin and total proteins by a Mindray BS-5800M automatic biochemical analyzer.

### Statistical Analysis of the Data

SPSS 20.0 software was used for the one-way ANOVA analysis and Tukey's test was used for multiple comparisons. The experimental data are expressed as the mean ± standard error of the mean (SEM). *P* < 0.05 was considered significant.

## Results

### Effects of Plectasin on the Performance of Yellow-Feathered Chickens

The average daily feed intake (ADFI), average daily gain (ADG), feed to gain ratio (F/G) and survival rate of yellow-feathered chickens were determined by different treatments on days 1–21, 22–42, 43–63 and 1–63 among group A (control group), group B (10 mg enramycin/kg of diet), and group C (100 mg plectasin/kg of diet) and group D (200 mg plectasin/kg of diet). The results are shown in Table 4.

**Table 4:** The growth performance of yellow-feathered chickens in different treatment groups.

**Stage**	**Index**	**Group A**	**Group B**	**Group C**	**Group D**
1–21 days	ADG/g	17.05 ± 0.26^c^	21.22 ± 0.30^ab^	20.44 ± 0.18^b^	21.45 ± 0.22^a^
	ADFI/g	30.11 ± 0.38^a^	29.36 ± 0.43^ab^	29.03 ± 0.31^b^	28.94 ± 0.41^b^
	F/G	1.77 ± 0.009^a^	1.40 ± 0.007^c^	1.44 ± 0.005^b^	1.35 ± 0.004^c^
	Survival rate (%)	96.80	98.67	98.40	98.93
22–42 days	ADG/g	30.71 ± 0.52^b^	38.72 ± 0.68^a^	37.55 ± 0.73^a^	37.98 ± 0.66^a^
	ADFI/g	76.69 ± 0.58	75.84 ± 1.01	75.91 ± 1.17	75.58 ± 1.17
	F/G	2.50 ± 0.030^a^	1.96 ± 0.028^c^	2.02 ± 0.024^b^	1.99 ± 0.021^b^
	Survival rate (%)	98.6	99.72	99.19	99.73
43–63 days	ADG/g	37.00 ± 1.31^d^	39.60 ± 0.96^c^	41.75 ± 0.63^b^	43.69 ± 0.73^a^
	ADFI/g	115.59 ± 2.63^a^	113.05 ± 1.45^b^	112.52 ± 1.57^b^	113.17 ± 1.9^b^
	F/G	3.12 ± 0.05^a^	2.86 ± 0.06^b^	2.70 ± 0.03^c^	2.59 ± 0.05^d^
	Survival rate (%)	98.04	98.37	98.36	99.19
1–63 days	ADG/g	27.78 ± 0.62^c^	30.22 ± 0.33^b^	32.25 ± 0.47^a^	32.62 ± 0.60^a^
	ADFI/g	74.38 ± 1.21^a^	71.32 ± 0.29^ab^	70.90 ± 0.83^b^	70.17 ± 1.37^b^
	F/G	2.68 ± 0.02^a^	2.36 ± 0.02^b^	2.20 ± 0.02^c^	2.15 ± 0.02^c^
	Survival rate (%)	93.60	96.80	96.00	97.87

*In the same row, values with different letter superscripts mean significant difference (P < 0.05), while with the same or no letter superscripts mean no significant difference (P > 0.05). Group A represents the basal diet; Group B represents basal diet supplemented with 10 mg enramycin/kg; Group C represents basal diet supplemented with 100 mg plectasin/kg; Group D represents basal diet supplemented with 200 mg plectasin/kg*.

As shown in Table 4, on days 1–21, the ADG of group D was significantly higher than those in group A and group C (*P* < 0.05), the ADFI in group D and group C was significantly lower than that in group A (*P* < 0.05), the F/G in group D and group C was significantly lower than that in group A (*P* < 0.05). Additionally, the ADG of group D was significantly higher than that in group C (*P* < 0.05), while the F/G of group D was significantly lower than that in group C (*P* < 0.05), indicating that administration of 200 mg plectasin/kg of diet is better than 100 mg plectasin/kg of diet. On days 22–42, the ADG of groups B, C, and D was significantly higher than that in group A (*P* < 0.05), while the F/G of groups B, C, and D was significantly lower than that in group A (*P* < 0.05). On days 43–63, the ADG of group D was significantly higher than that in groups A, B, and C (*P* < 0.05) and the ADFI in groups B, C, and D was significantly lower than that in group A (*P* < 0.05). Furthermore, the F/G was significantly lower in groups C and D than in groups A and B (*P* < 0.05). For the overall 63-day period, the ADG was significantly higher in groups C and D than in groups A and B, while the ADFI was significantly lower in groups C and D than in group A (*P* < 0.05). Furthermore, the F/G was significantly lower in groups C and D than in group A (*P* < 0.05).

### Effects of Plectasin on the Antibody Titer of the H9N2 Influenza Virus and Newcastle Disease Virus in Yellow-Feathered Chickens

Tables 5, 6 show the serum antibody levels of each stage of yellow-feathered chickens after immunization with the H9N2 AIV and NDV vaccines in group A (control group), group B (10 mg enramycin/kg of diet), group C (100 mg plectasin/kg of diet), and group D (200 mg plectasin/kg of diet).

**Table 5:** Serum H9N2 AIV antibody levels of yellow-feathered chickens in different treatment groups.

**Group**	**A**	**B**	**C**	**D**
7 days of age	9.81 ± 0.20	9.83 ± 0.49	9.85 ± 0.20	9.86 ± 0.40
21 days of age	6.24 ± 0.20^b^	6.26 ± 0.24^b^	6.53 ± 0.24^a^	6.59 ± 0.20^a^
35 days of age	7.29 ± 0.73^b^	7.32 ± 0.58^b^	8.02 ± 0.40^ab^	8.75 ± 1.03^a^
42 days of age	9.41 ± 0.24^c^	9.97 ± 0.37^b^	10.18 ± 0.20^b^	11.53 ± 0.24^a^

*In the same row, values with different letter superscripts mean significant difference (P < 0.05), while with the same or no letter superscripts mean no significant difference (P > 0.05). Group A represents the basal diet; Group B represents basal diet supplemented with 10 mg enramycin/kg; Group C represents basal diet supplemented with 100 mg plectasin/kg; Group D represents basal diet supplemented with 200 mg plectasin/kg*.

**Table 6:** Serum NDV antibody levels of yellow-feathered chickens in different treatment groups.

**Group**	**A**	**B**	**C**	**D**
7 days of age	8.78 ± 0.20	8.84 ± 0.37	9.00 ± 0.32	9.10 ± 0.48
21 days of age	6.40 ± 0.24^c^	6.85 ± 0.29^bc^	7.00 ± 0.55^b^	7.61 ± 0.25^a^
35 days of age	7.60 ± 0.68^b^	8.25 ± 0.75^b^	8.40 ± 0.60^b^	9.50 ± 0.29^a^
42 days of age	9.04 ± 0.32^a^	9.48 ± 0.51^a^	9.59 ± 0.65^a^	9.74 ± 0.40^a^

*In the same row, values with different letter superscripts mean significant difference (P < 0.05), while with the same or no letter superscripts mean no significant difference (P > 0.05). Group A represents the basal diet; Group B represents basal diet supplemented with 10 mg enramycin/kg; Group C represents basal diet supplemented with 100 mg plectasin/kg; Group D represents basal diet supplemented with 200 mg plectasin/kg*.

As shown in Table 5, serum antibody levels of H9N2 AIV were significantly higher in group D than in groups A and B at 21 and 35 days of age (*P* < 0.05). Besides, the serum antibody level of H9N2 AIV was significantly higher in group D than in groups A, B, and C at 42 days of age (*P* < 0.05).

As shown in Table 6, the serum antibody level of NDV was significantly higher in group D than in groups A, B, and C at 21 and 35 days of age (*P* < 0.05). Additionally, the serum antibody level of NDV was significantly higher in group D than in group C at 21 and 35 days of age (*P* < 0.05).

### Effects of Plectasin on the Intestinal Morphology and Structure in Yellow-Feathered Chickens

The villus lengths, crypt depths, and villus length/crypt depth ratio of the ileum, jejunum and duodenum of 21-day-old yellow-feathered chickens in group A (control group), group B (10 mg enramycin/kg of diet), group C (100 mg plectasin/kg of diet), and group D (200 mg plectasin/kg of diet) are shown in Table 7.

**Table 7:** Villus length, crypt depth and villus length/crypt depth of 21-day-old yellow -feathered chickens in different treatment groups.

**Site**	**Index**	**Group A**	**Group B**	**Group C**	**Group D**
Ileum	Villus length	0.659 ± 0.007^d^	0.713 ± 0.008^b^	0.685 ± 0.004^c^	0.740 ± 0.002^a^
	Crypt depth	0.152 ± 0.002^a^	0.142 ± 0.002^c^	0.147 ± 0.002^b^	0.137 ± 0.001^d^
	Villus length/crypt depth	4.337 ± 0.080^d^	5.035 ± 0.060^b^	4.825 ± 0.067^c^	5.205 ± 0.075^a^
Jejunum	Villus length	0.953 ± 0.002^c^	0.959 ± 0.003^bc^	0.966 ± 0.006^b^	0.981 ± 0.001^a^
	Crypt depth	0.165 ± 0.003^a^	0.158 ± 0.001^b^	0.156 ± 0.003^b^	0.150 ± 0.002^c^
	Villus length/crypt depth	5.778 ± 0.114^c^	6.070 ± 0.049^b^	6.194 ± 0.109^b^	6.541 ± 0.083^a^
Duodenum	Villus length	1.134 ± 0.001^c^	1.137 ± 0.002^c^	1.168 ± 0.004^b^	1.191 ± 0.001^a^
	Crypt depth	0.254 ± 0.003^a^	0.244 ± 0.001^b^	0.236 ± 0.001^c^	0.227 ± 0.002^d^
	Villus length/crypt depth	4.465 ± 0.051^d^	4.660 ± 0.020^c^	4.949 ± 0.018^b^	5.247 ± 0.061^a^

*Unit: mm*.

*In the same row, values with different letter superscripts mean significant difference (P < 0.05), while with the same or no letter superscripts mean no significant difference (P > 0.05). Group A represents the basal diet; Group B represents basal diet supplemented with 10 mg enramycin/kg; Group C represents basal diet supplemented with 100 mg plectasin/kg; Group D represents basal diet supplemented with 200 mg plectasin/kg*.

**Figure 1:**
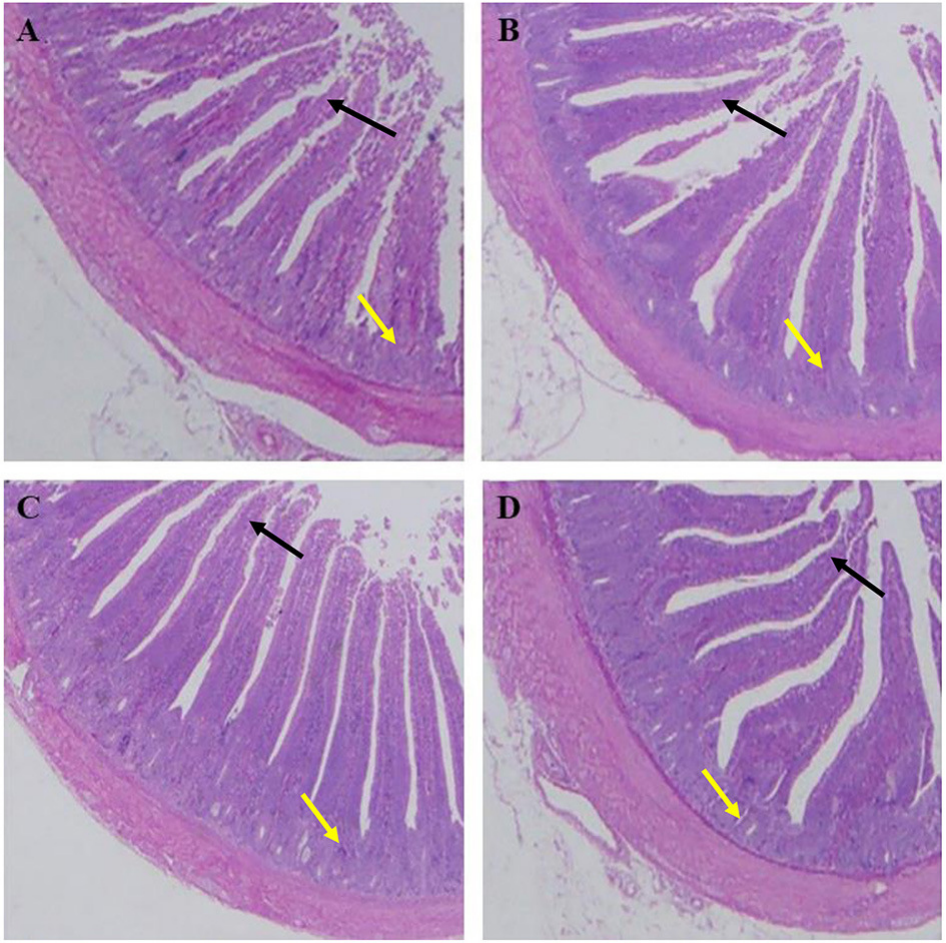
Dietary plectasin increased the ileum villus length while decreased crype depth of 21-day-old yellow-feathered chickens. **(A)** The ileum of basal diet group, **(B)** The ileum of basal diet plus 10 mg enramycin/kg, **(C)** The ileum of basal diet plus 100 mg/kg plectasin, **(D)** The ileum of basal diet plus 200 mg/kg plectasin. Black arrow shows the villus length, and yellow arrow shows the crypt depth. For histological observation, image at 40 were provided.

**Figure 2:**
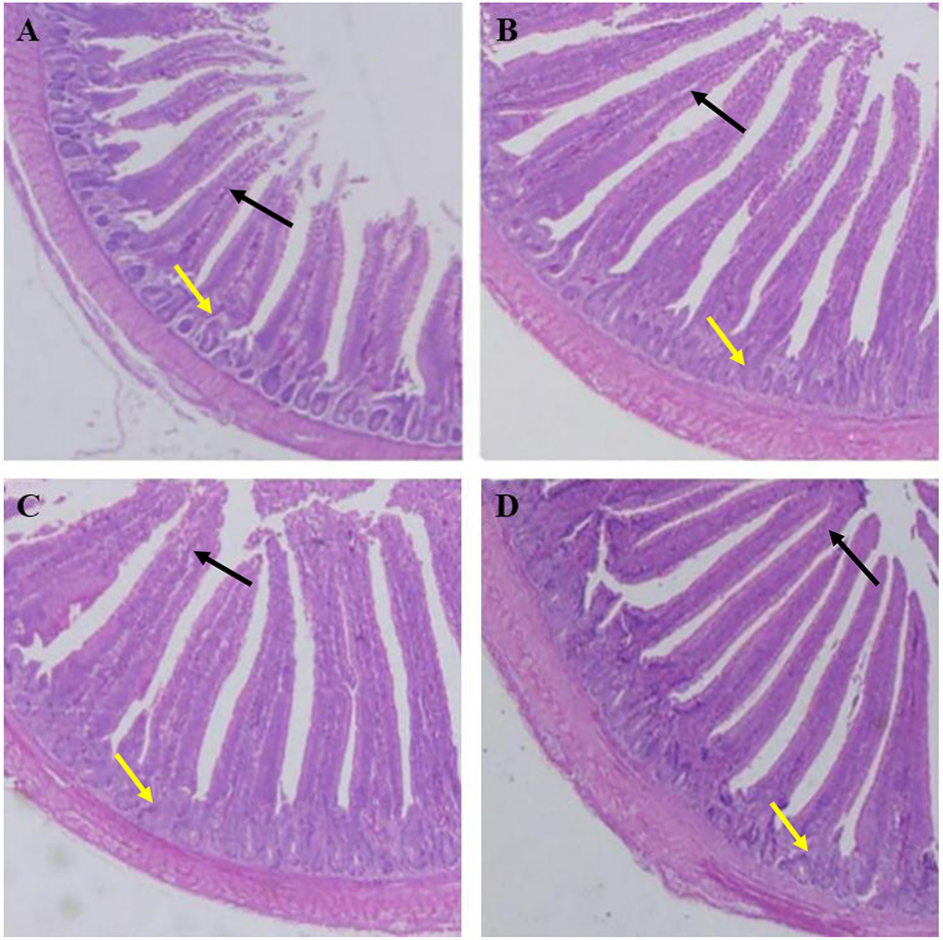
Dietary plectasin increased the jejunum length while decreased crype depth of 21-day-old yellow-feathered chickens. **(A)** The jejunum of basal diet group, **(B)** The jejunum of basal diet plus 10 mg enramycin/kg, **(C)** The jejunum of basal diet plus 100 mg/kg plectasin, **(D)** The jejunum of basal diet plus 200 mg/kg plectasin. Black arrow shows the villus length, and yellow arrow shows the crypt depth. For histological observation, image at 40 were provided.

**Figure 3:**
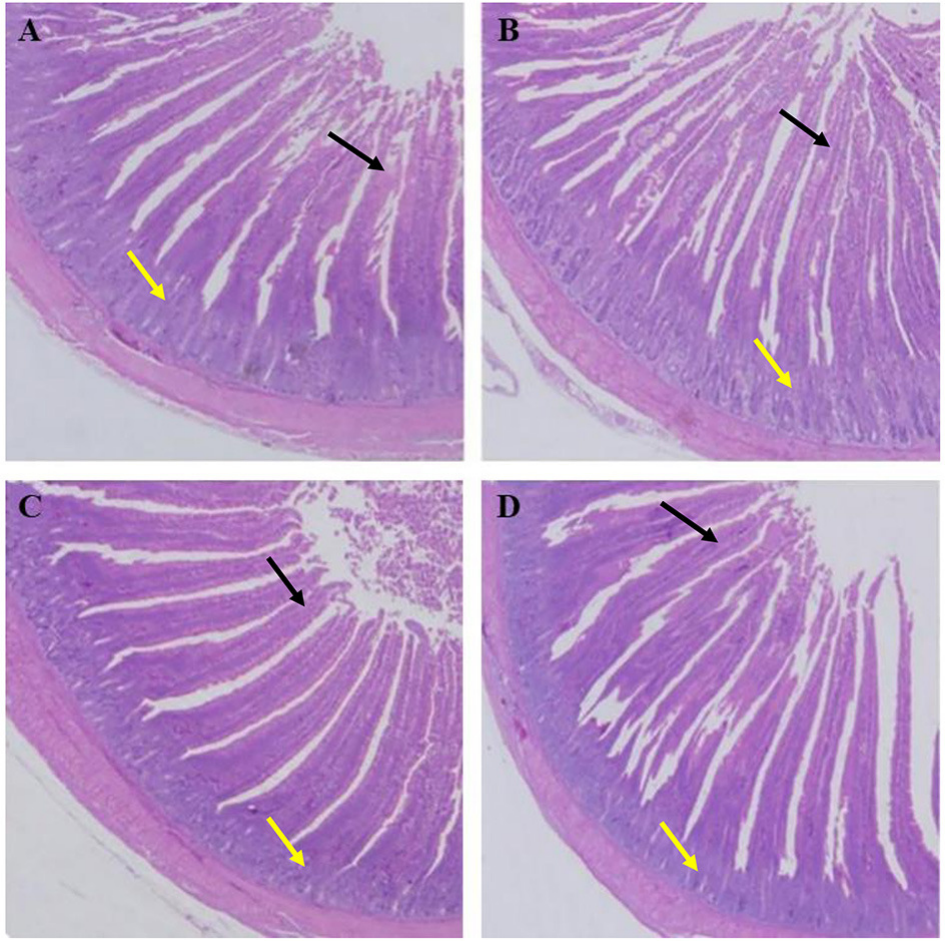
Dietary plectasin increased the duodenum length while decreased crype depth of 21-day-old yellow-feathered chickens. **(A)** The duodenum of basal diet group, **(B)** The duodenum of basal diet plus 10 mg enramycin/kg, **(C)** The duodenum of basal diet plus 100 mg/kg plectasin, **(D)** The duodenum of basal diet plus 200 mg/kg plectasin. Black arrow shows the villus length, and yellow arrow shows the crypt depth. For histological observation, image at 40 were provided.

As shown in Table 7, the villus lengths of the ileum, jejunum, and duodenum were significantly greater in group D than in groups A, B, and C (*P* < 0.05); the crypt depths of the ileum, jejunum and duodenum were significantly lower in group D than in groups A, B, and C (*P* < 0.05), and the villus length/crypt depth of the ileum, jejunum, and duodenum was significantly greater in group D than in groups A, B, and C (*P* < 0.05). Tissue sections of the ileum, jejunum, and duodenum are shown in Figures 1–3, respectively.

### Effects of Plectasin on the Intestinal Microflora in Yellow-Feathered Chickens

**Figure 4:**
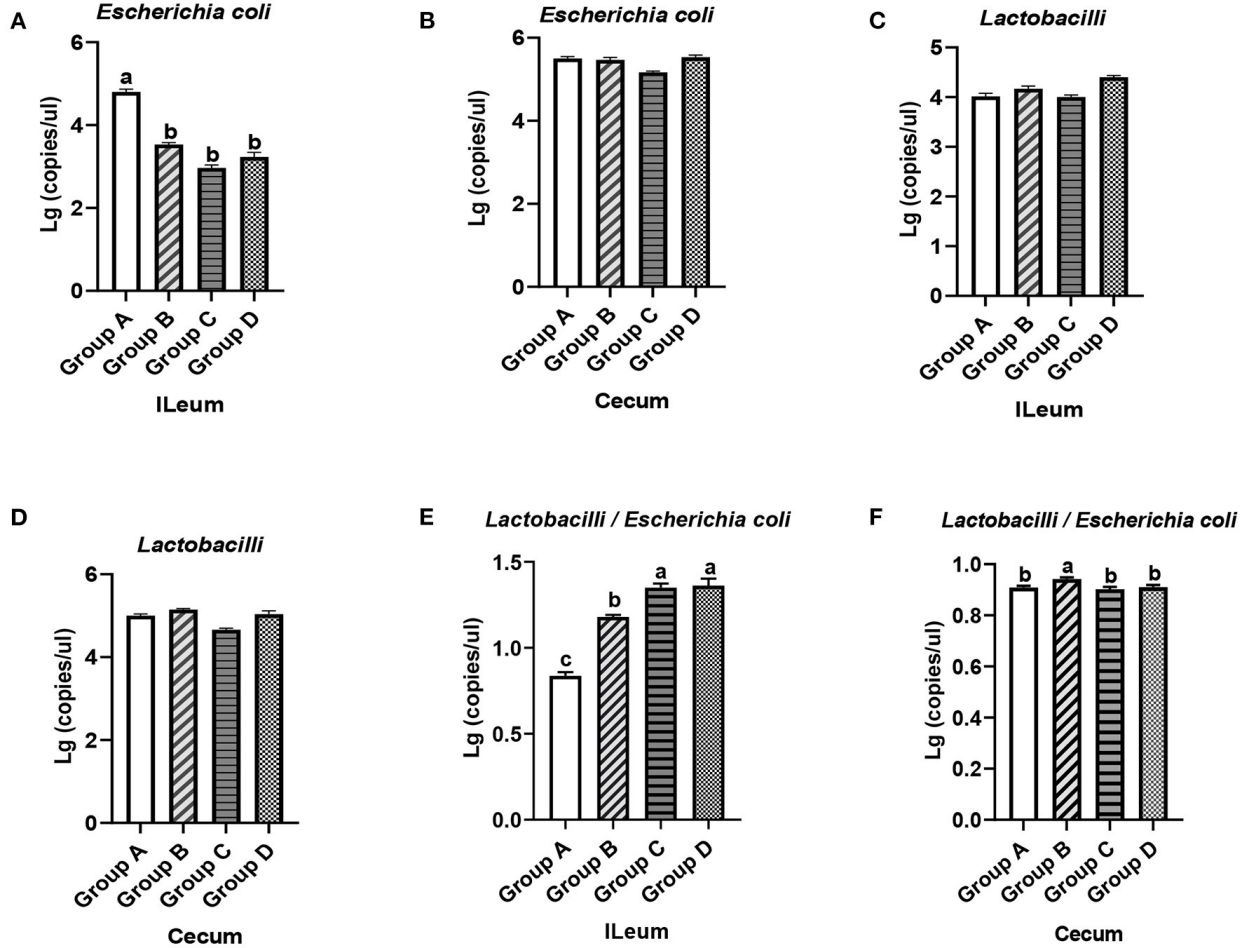
Quantity of *E. coli* and *Lactobacilli* in the ileums and ceca of 21-day-old yellow-feathered chickens in different treatment groups determined by absolute quantitative PCR. **(A)** Dietary plectasin could significantly decrease the quantity of *E. coli* in the ileal contents of 21-day-old yellow-feathered chickens. **(B)** Dietary plectasin had no significant effect on the quantity of *E. coli* in the cecal contents of 21-day-old yellow-feathered chickens. **(C)** Dietary plectasin had no significant effect on the quantity of *Lactobacilli* in the ileal contents of 21-day-old yellow-feathered chickens. **(D)** Dietary plectasin had no significant effect on the quantity of *Lactobacilli* in the cecal contents of 21-day-old yellow-feathered chickens. **(E)** Dietary plectasin could significantly increase the ratio between *Lactobacilli* and *E. coli* in the ileal contents of 21-day-old yellow-feathered chickens. **(F)** Dietary plectasin had no significant effect on the ratio between *Lactobacilli* and *E. coli* in the cecal contents of 21-day-old yellow-feathered chickens. Superscripts of the same letter indicate that there was no significant difference (*P* > 0.05), and superscripts of different letters indicate significant differences (*P* < 0.05). Data are presented as the mean ± standard error of mean (SEM) of three independent experiments. The differences between groups were analyzed using one-way ANOVA and Tukey's test. Group A: control group; group B: 10 mg enramycin/kg of diet; group C: 100 mg plectasin/kg of diet; group D: 200 mg plectasin/kg of diet.

The effects of plectasin on the intestinal microflora of 21-day-old yellow-feathered chickens from group A (control group), group B (10 mg enramycin/kg of diet), group C (100 mg plectasin/kg of diet), and group D (200 mg plectasin/kg of diet) are shown in Figure 4.

As can be seen from Figure 4A, compared with the group A, the content of *E. coli* in the ileums of chickens from groups B, C, and D significantly decreased (*P* < 0.05). In addition, the content of *E. coli* in the ceca of chickens in groups B, C, and D was not significantly changed compared with that of group A (*P* > 0.05) (Figure 4B). As shown in Figures 4C,D, there was no significantly change in the *Lactobacilli* concentration in the ileums and ceca of chickens in groups A, B, C, and D (*P* > 0.05). As shown in Figure 4E, in the ileum, the ratio between *Lactobacilli* and *E. coli* was significantly higher in groups C and D than in groups A and B (*P* < 0.05), while the ratio between *Lactobacilli* and *E. coli* was significantly higher in the ceca of chickens in group B than in groups A, C, and D (Figure 4F). The above results show that dietary plectasin can cause intestinal flora changes in yellow-feathered chickens through reducing the concentration of *E. coli* in the ileum while increasing the ratio of between *Lactobacilli* and *E. coli* in the ileum.

### Effects of Plectasin on Mucosal Layer Construction and Tight Junctions of Ileum of Yellow-Feathered Chickens

**Figure 5:**
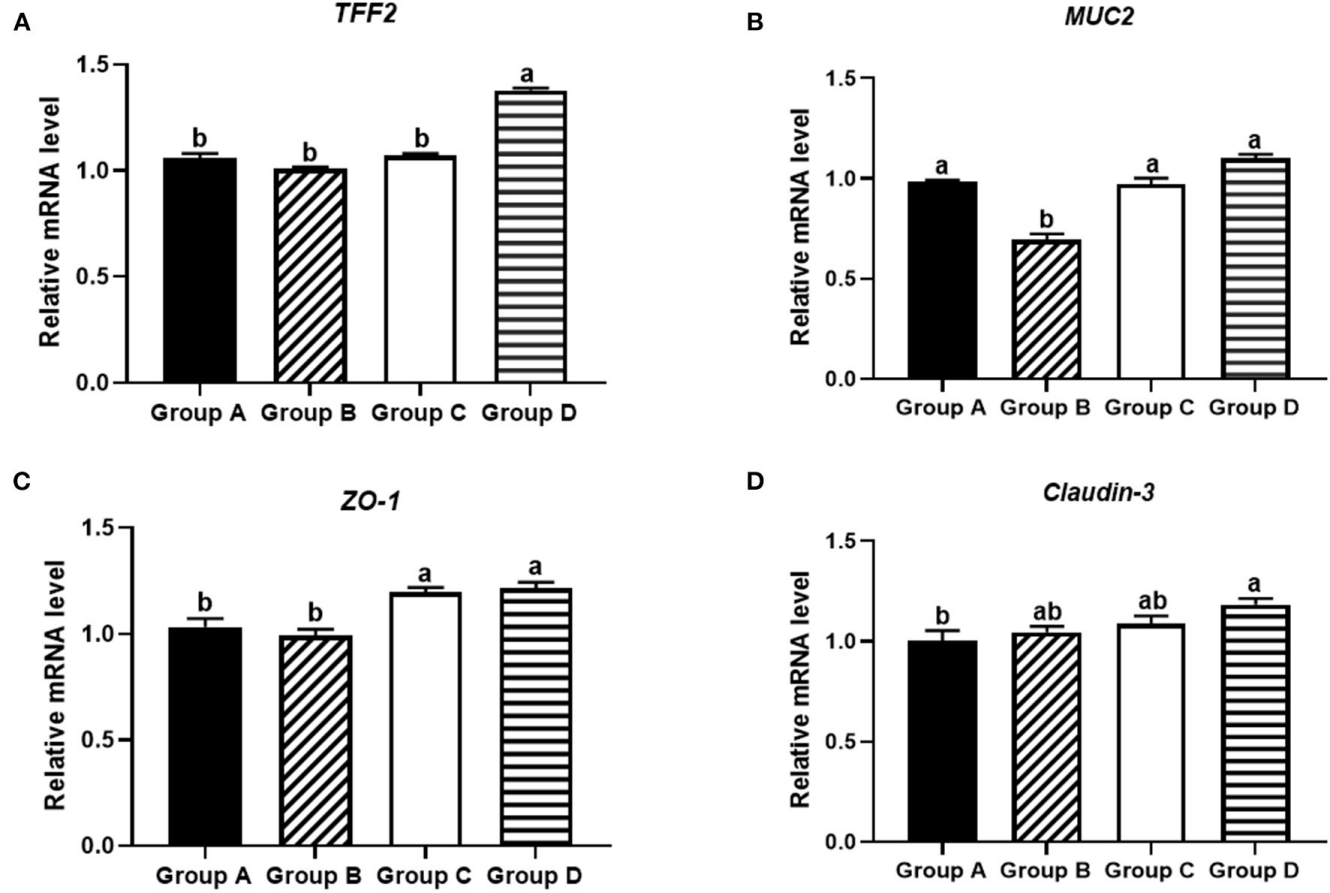
Effects of different treatments on tight junction proteins in the ileums of 21-day-old yellow-feathered chickens in different treatment groups determined by absolute quantitative PCR. **(A)** Dietary plectasin could significantly increase the mRNA expression level of *TFF2* in the ileums of 21-day-old yellow-feathered chickens, as found by qRT-PCR. **(B)** Dietary plectasin had no significantly effect on the expression level of *MUC2* in the ileums of 21-day-old yellow-feathered chickens, as found by qRT-PCR. **(C)** Dietary plectasin could significantly increase the mRNA expression level of *ZO-1* in the ileums of 21-day-old yellow-feathered chickens, as found by qRT-PCR. **(D)** Dietary plectasin could significantly increase the mRNA expression level of *Claudin-3* in the ileums of 21-day-old yellow-feathered chickens, as found by qRT-PCR. Superscripts of the same letter indicate no significant difference (*P* > 0.05), and the superscripts of different letters indicate significant differences (*P* < 0.05). Data are presented as the mean ± standard error of mean (SEM) of three independent experiments. The differences between groups were analyzed using one-way ANOVA and Tukey's test. Group A: control group; group B: 10 mg enramycin/kg of diet; group C: 100 mg plectasin/kg of diet; group D: 200 mg plectasin/kg of diet.

The effects of plectasin on the mucosal layer construction and tight junctions of ileum of 21-day-old yellow-feathered chickens from group A (control group), group B (10 mg enramycin/kg of diet), group C (100 mg plectasin/kg of diet), and group D (200 mg plectasin/kg of diet) are shown in Figure 5.

As shown in Figure 5A, at 21 days of age, the mRNA expression of *TFF2* was significantly higher in group D than in other groups (*P* < 0.05). As shown in Figure 5B, the mRNA expression of *MUC2* was significantly higher in groups A, C, and D than in group B (*P* < 0.05). As shown in Figure 5C, the mRNA expression of *ZO-1* in groups C and D was significantly higher than in groups A and B (*P* < 0.05). As shown in Figure 5D, the mRNA expression of *Claudin-3* was significantly higher in group D than in group A (*P* < 0.05).

### Effects of Plectasin on Ileal Immune Factors in Yellow-Feathered Chickens

**Figure 6:**
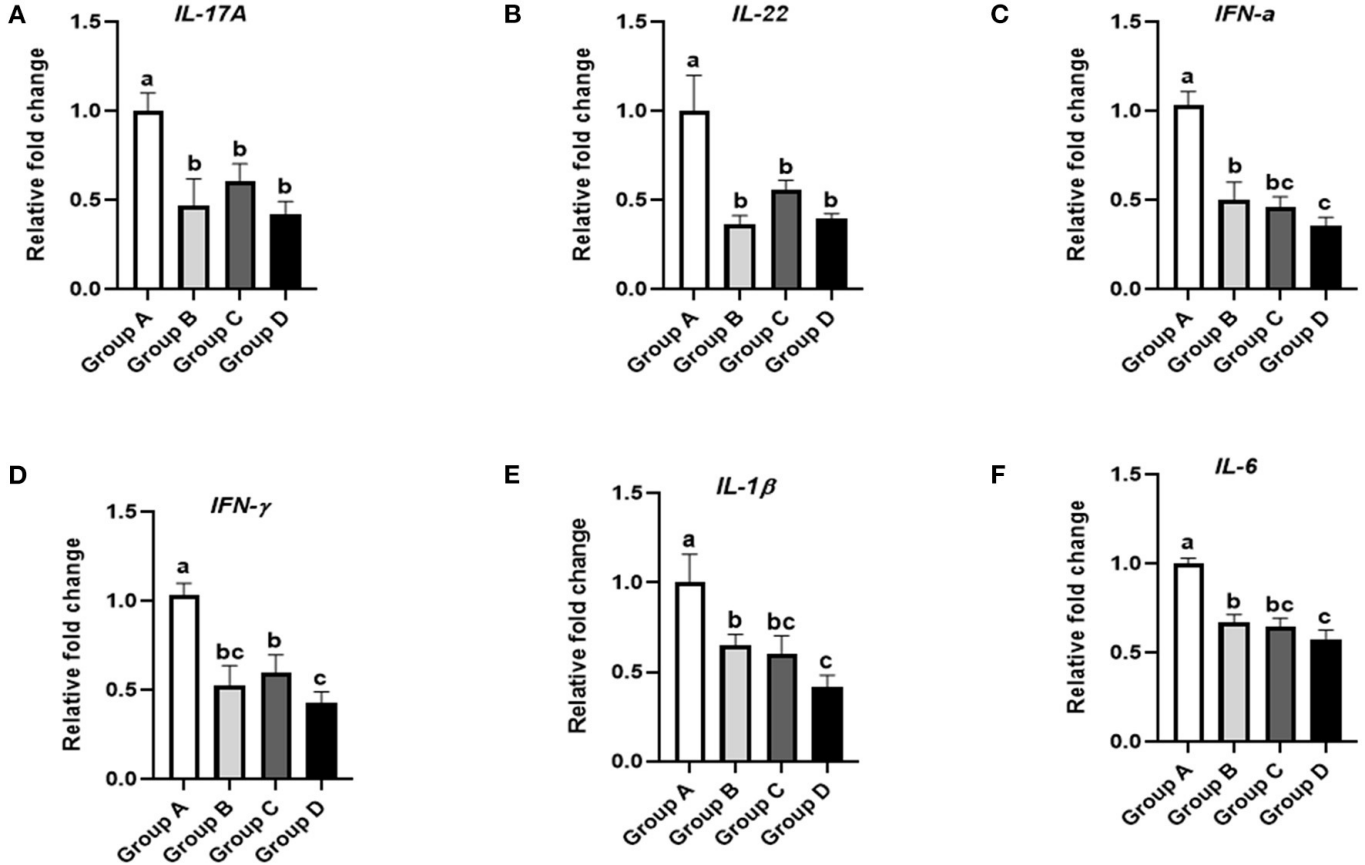
Effects of different treatments on the ileal immune factors of 21-day-old yellow-feathered chickens. **(A)** Dietary plectasin could significantly decrease the mRNA expression level of *IL-17A* in the ileums *of* 21-day-old yellow-feathered chickens, as found by qRT-PCR. **(B)** Dietary plectasin could significantly decrease the mRNA expression level of *IL-22* in the ileums of 21-day-old yellow-feathered chickens, as found by qRT-PCR. **(C)** Dietary plectasin could significantly decrease the mRNA expression level of *IFN-*α in the ileums of 21-day-old yellow-feathered chickens, as found by qRT-PCR. **(D)** Dietary plectasin could significantly decrease the mRNA expression level of *IFN-*γ in the ileums of 21-day-old yellow-feathered chickens, as found by qRT-PCR. **(E)** Dietary plectasin could significantly decrease the mRNA expression level of *IL-1*β in the ileums of 21-day-old yellow-feathered chickens, as found by qRT-PCR. **(F)** Dietary plectasin could significantly decrease the mRNA expression level of *IL-6* in the ileums of 21-day-old yellow-feathered chickens, as found by qRT-PCR. Superscripts of the same letter indicate no significant difference (*P* > 0.05), and superscripts of different letters indicate significant differences (*P* < 0.05). Data are presented as the mean ± standard error of mean (SEM) of three independent experiments. The differences between groups were analyzed using one-way ANOVA and Tukey's test. Group A: control group; group B: 10 mg enramycin/kg of diet; group C: 100 mg plectasin/kg of diet; group D: 200 mg plectasin/kg of diet.

The effects of plectasin on the ileal immune factors *IL-17A, IL-22, IFN-*α, *IFN-*γ*, IL-1*β, and *IL-6* in 21-day-old yellow-feathered chickens from group A (control group), group B (10 mg enramycin/kg of diet), group C (100 mg plectasin/kg of diet), and group D (200 mg plectasin/kg of diet) are shown in Figure 6.

As shown in Figure 6A, *IL-17A* expression in the ileum was significantly decreased in groups B, C, and D compared with group A (*P* < 0.05). As shown in Figure 6B, the expression of *IL-22* in the ileum was significantly decreased in groups B, C, and D compared with group A (*P* < 0.05). As shown in Figure 6C, *IFN-*α expression in the ileum was significantly decreased in groups B, C, and D compared with group A (*P* < 0.05). Additionally, *IFN-*α expression in the ileum was significantly lower in group D than in group B (*P* < 0.05). As shown in Figure 6D, *IFN-*γ expression in the ileum was significantly decreased in groups B, C, and D compared with group A (*P* < 0.05). As shown in Figure 6E, *IL-1*β expression in the ileum was significantly lower in groups B, C, and D compared with group A (*P* < 0.05). Furthermore, *IL-1*β expression in the ileum was significantly lower in group D than in group B (*P* < 0.05). As shown in Figure 6F, *IL-6* expression in the ileum was significantly lower in groups B, C, and D than in group A (*P* < 0.05). Moreover, *IL-6* expression in the ileum was significantly lower in group D than in group B (*P* < 0.05).

### Effects of Plectasin on Blood Biochemical Indices in Yellow-Feathered Chickens

The effects of plectasin on blood biochemical indices in 21-day-old yellow-feathered chickens in chickens from group A (control group), group B (10 mg enramycin/kg of diet), group C (100 mg plectasin/kg of diet), and group D (200 mg plectasin/kg of diet) are shown in Table 8.

**Table 8:** Blood biochemical indices of 21-day-old yellow-feathered chickens in different treatment groups.

	**Group A**	**Group B**	**Group C**	**Group D**
Glucose (mmol/L)	14.891 ± 0.03^c^	14.987 ± 0.07^c^	15.523 ± 0.08^b^	16.093 ± 0.05^a^
Triglyceride (g/L)	2.724 ± 0.04^b^	2.960 ± 0.02^a^	2.877 ± 0.05^a^	2.983 ± 0.02^a^
Total cholesterol (g/L)	18.070 ± 0.03^c^	18.641 ± 0.04^b^	18.653 ± 0.01^b^	19.032 ± 0.04^a^
High-densitycholesterol (g/L)	3.696 ± 0.01	3.787 ± 0.03	3.772 ± 0.06	3.833 ± 0.04
Total proteins (g/L)	20.523 ± 0.04^b^	20.403 ± 0.02^b^	20.444 ± 0.05^b^	20.982 ± 0.04^a^
Albumin (g/L)	11.687 ± 0.04^b^	12.093 ± 0.06^a^	11.687 ± 0.05^b^	11.743 ± 0.03^b^

*In the same row, values with different letter superscripts mean significant difference (P < 0.05), while with the same or no letter superscripts mean no significant difference (P > 0.05). Group A represents the basal diet; Group B represents basal diet supplemented with 10 mg enramycin/kg; Group C represents basal diet supplemented with 100 mg plectasin/kg; Group D represents basal diet supplemented with 200 mg plectasin/kg*.

As shown in Table 8, the glucose concentration was significantly higher in group D than in groups A, B, and C (*P* < 0.05). The triglyceride concentration was significantly higher in groups B, C, and D than in group A (*P* < 0.05). The total cholesterol concentration was significantly higher in groups B, C, and D than in group A (*P* < 0.05). Meanwhile, the total cholesterol was significantly higher in group D than in groups A, B, and C (*P* < 0.05). However, there was no significant difference in the high-density cholesterol concentration among the different groups (*P* > 0.05). The albumin concentration was significantly higher in group B than in groups A, C, and D (*P* < 0.05). The total protein content was significantly higher in group D than in groups A, B, and C (*P* < 0.05). Additionally, the total protein content was significantly higher in group D than in group C (*P* < 0.05).

## Discussion

AMPs have good potential as suitable alternatives to the conventional antibiotics used in swine and poultry industries (39). It was reported that AMPs have beneficial effects on growth performance, reducing the incidence of diarrhea and increasing the rate of weaned pigs (40). It was also reported that the bioactive peptides derived from cottonseed (BPC) could induce favorable influences on growth performance, immune responses and total antioxidant activity of serum in broiler chickens (41, 42). Additionally, Daneshmand et al. (18) showed that the AMPs could protect broilers against *in vivo* challenging *E. coli*. A previous study showed that plectasin elicits similar improvements in performance and intestinal mucosa growth and activity in weaned pigs when used as an antibiotic (43). However, there has been no research on the growth performance of yellow-feathered chickens treated with plectasin. Hence, this study for the first time reveals that dietary plectasin could increase ADG while decreasing the F/G ratio for an overall period of 1–63 days. This may be because plectasin could enhance the intestinal barrier and immune function and promote chickens to digest and absorb nutrients.

Cellular immunity and humoral immunity are two important immune responses in animals. Antibodies are secreted by plasma cells and used by the immune system to recognize and neutralize foreign substances such as bacteria and viruses (44). The antibody titer in serum reflects the strength of the body's humoral immune function and the body's health under certain conditions (45). A previous study showed that the artificial antimicrobial peptide KLKLLLLLKLK predominantly induces a TH2-type immune response to coinjected antigens (46). In this study, after oral administration of plectasin, we speculated that plectasin can be absorbed by the intestinal tract and improve intestinal health. Some studies have shown that the improvement of intestinal health can improve the immunity of chickens (47, 48), so we speculated that the improvement of immunity of chickens plays a certain role in improving humoral immunity. The level of the antibody titer produced by the body receiving the antigen reflects the level of the chicken's specific humoral immune function. Theoretically, plectasin can generate specific and non-specific immunity in the intestine through the proliferation of beneficial bacteria, and then improve the body's cellular and humoral immunity through T lymphocytes and B lymphocytes. After being metabolized by the animal, the serum contains active ingredients that promote the proliferation of spleen lymphocytes, which can exert its immune regulation function, and significantly increased the antibody titer, thereby improving the protection rate of the vaccine to the animal and has the effect of preventing and controlling diseases.

The small intestine is an important place for digestion and absorption of the animal body, so the morphological structure of the small intestine is often used as an indicator to measure the digestion and absorption functions of an animal. The length of the villi and the depth of crypts reflect the effective area and function of intestinal digestion and absorption of nutrients, and their ratio comprehensively reflects the functional status of the small intestine (49): the larger the ratio, the larger the contact area of the intestinal mucosa with nutrients and the stronger digestion and absorption functions. In this study, we showed that dietary antimicrobial peptide plectasin (200 mg plectasin/kg of diet) could increase the ratio between the villus length and crypt depth of the ileum, jejunum, and duodenum. Previous study has shown that dietary plectasin could improve the jejunal morphology in broiler chickens (Arbor Acres) (23). The reduced ratio between the villus length and crypt depth is a crucial indicator of gut morphology alterations as it reflects in reduced surface area (50). The possible mechanism of plectasin (200 mg plectasin/kg of diet) for increasing the ratio between the villus length and crypt depth of the ileum, jejunum, and duodenum may be that the dietary antimicrobial peptide plectasin has the function of protecting and promoting the repair of intestinal villi, and is conducive to the growth of intestinal epithelial cells, thus enlarging the absorption area and improving the absorption efficiency of nutrients, as well as facilitating the synthesis of important substances.

Tight junction proteins in intestinal epithelial cells play a key role in the intestinal mucosal barrier, and the destruction of these proteins can lead to an increase in cell-to-cell permeability (51). A previous study showed that oral administration of antimicrobial peptide such as CL36 and cLFchimera could improve the abundance of tight junction proteins in chickens (18, 19). The results in this study indicated that antimicrobial peptide plectasin could maintain the integrity of the intestinal physical barrier by up-regulating the expression of *TFF-2, ZO-1*, and *Claudin-3* to stabilize the structural distribution of tight junction proteins, thereby improving the permeability of the colonic mucosal barrier, suggesting that its potential role in promoting intestinal function and growth performance.

The intestinal microecological flora of chickens plays an important role in immune regulation and disease control. Intestinal microbes can be divided into intestinal symbiotic bacteria, conditional pathogenic bacteria, and pathogenic bacteria regarding to the relationship between the intestinal tract and host (52). The intestinal tract concentrations of conditioned pathogens are low, such as *E. coli* which is facultative anaerobes, but when intestinal homeostasis is disrupted, for instance, when the body is infected by virus, the proliferation of *E. coli* is rapidly promoted and led to intestinal disorders (53). In this study, application of the dietary antimicrobial peptide plectasin can significantly reduce the number of *E. coli* in the ileum and promote an increase in the content of *Lactobacillus* in the ileum, illustrating that plectasin can inhibit the proliferation of harmful bacteria in the intestine and change the structure of intestinal flora. However, the specific mechanism by which this occurs still needs further study. On possible reason for increasing the *Lactobacillus* population in the intestinal of chicken is due to decreasing the pathogenic population such as *E. coli* and in this condition the *Lactobacillus* have more opportunity to grow. It seems, the peptide in higher dose (200 mg) likely can escape more from intestinal digestion and can direct antibacterial effects on *E. coli*. May be the peptide have more antibacterial effects on Gram negative bacteria vs. Gram positive bacteria for yellow-feathered chickens.

Cytokines play an important role in the immune response. Some studies indicated that AMPs have an important effect on NF-kB pathway regarding immunomodulatory activities of AMPs (54, 55). Besides, AMPs plays a crucial role in immune regulation by activating the mitogen-activated protein kinase signaling pathway (56). The expression of proinflammatory cytokines in the body is abnormal when disease occurs, causing pathological damage and immune function lessening. Both *IL-17A* and *IL-22* are representative cytokines secreted by Th17 cells. *IL-17A* is an inflammatory cytokine that can promote the activation of T cells and stimulate epithelial cells to produce a variety of inflammatory factors (such as *IL-6* etc.). Both *IFN-*α and *IFN-*γ play important anti-infection and immune regulation roles. Our study showed that the expression of interferon was positively correlated with the degree of inflammation *in vivo* of the intestine, and dietary plectasin can significantly decrease the mRNA expression levels of *IFN-*α and *IFN-*γ in the ileums of yellow-feathered chickens at 21 days of age. In this study, plectasin may maintain intestinal homeostasis by inhibiting the production of pro-inflammatory cytokines, reducing inflammatory cell infiltration, repairing intestinal barrier damage to a certain extent, enhancing intestinal epithelial barrier function, thereby reducing intestinal inflammation and achieving a protective effect on the intestinal tract.

Serum biochemical indices were commonly used as a standard to measure animal health because that they can reflect the physiological state and health of the animals (57). A large number of studies have demonstrated that antimicrobial peptides could affect the serum biochemical indices in *Epinephelus coioides*, weaned piglets, and chickens (58, 59). To a certain extent, blood biochemical indices could reflect the body's absorption and utilization of nutrients. For example, glucose is an important energy source of animal body, and the decrease of glucose content may be closely related to abnormal glucose metabolism, severe liver disease, malnutrition (60). The decrease of serum triglyceride is commonly associated with impaired liver function and malnutrition (61). Serum total cholesterol is mainly synthesized by the liver and is the main component that synthesizes a variety of hormones and forms the cell membrane (62). As a reflection of protein metabolism and immune function, the level of serum total protein can reflect the strength of protein synthesis and metabolism (61). In this study, pletasin is beneficial to improve the disorder of lipid metabolism and increase feed conversion rate of broilers to a certain extent by changing the contents of serum glucose, triglyceride and total cholesterol. Feeding the antimicrobial peptide for a long time could have the effects of disease prevention and resistance, which is conducive to the body health, ensures the health and production performance, and further improves the quality of meat products.

In summary, production performance, measured as the ADG, was improved while F/G was decreased after the addition of plectasin to feed compared with the addition of enramycin over a period of 63 days. Dietary plectasin intake can enhance the antibody levels of H9N2 AIV and NDV in yellow-feathered chickens at 21 and 35days of age. Dietary plectasin can enhance the intestine structure, inhibit *E. coli* and proinflammatory cytokines in the ileum, and ameliorate blood biochemical indices in yellow-feathered chickens.

## Data Availability Statement

The raw data supporting the conclusions of this article will be made available by the authors, without undue reservation.

## Ethics Statement

The animal study was reviewed and approved by Animal Care Committee of South China Agricultural University (approval ID: SYXK-2014-0136).

## Author Contributions

QX and FenC conceived and designed the study. ZYao, ZYan, FeiC, and ZX collected the samples. CW, LW, RL, and LC conducted laboratory analysis and acquired the data. XZ and QZ conducted bioinformatics, data analysis, and drafted the manuscript. LW analyzed the data and provided suggestions on the design of the experiment. All authors contributed to the article, critically revised the manuscript, and approved the submitted version.

## Conflict of Interest

LW was employed by Guangdong Hinabiotech Co., Ltd. ZYan was employed by Wen's Foodstuff Group Co., Ltd. The remaining authors declare that the research was conducted in the absence of any commercial or financial relationships that could be construed as a potential conflict of interest.
